# Proteomic Analysis of Proteins Related to Defense Responses in *Arabidopsis* Plants Transformed with the *rolB* Oncogene

**DOI:** 10.3390/ijms24031880

**Published:** 2023-01-18

**Authors:** Yulia V. Vereshchagina, Anastasiya A. Mironova, Dmitry V. Bulgakov, Victor P. Bulgakov

**Affiliations:** Federal Scientific Center of the East Asia Terrestrial Biodiversity, Far East Branch of Russian Academy of Sciences, 159 Stoletija Str., 690022 Vladivostok, Russia

**Keywords:** *Arabidopsis*, *rolB* oncogene, plant defense, plant–microbe interaction, proteomics, PYK10 complex, RACK1A

## Abstract

During *Agrobacterium rhizogenes*–plant interaction, the *rolB* gene is transferred into the plant genome and is stably inherited in the plant’s offspring. Among the numerous effects of *rolB* on plant metabolism, including the activation of secondary metabolism, its effect on plant defense systems has not been sufficiently studied. In this work, we performed a proteomic analysis of *rolB*-expressing *Arabidopsis thaliana* plants with particular focus on defense proteins. We found a total of 77 overexpressed proteins and 64 underexpressed proteins in *rolB-*transformed plants using two-dimensional gel electrophoresis and MALDI mass spectrometry. In the *rolB*-transformed plants, we found a reduced amount of scaffold proteins RACK1A, RACK1B, and RACK1C, which are known as receptors for activated C-kinase 1. The proteomic analysis showed that *rolB* could suppress the plant immune system by suppressing the RNA-binding proteins GRP7, CP29B, and CP31B, which action are similar to the action of type-III bacterial effectors. At the same time, *rolB* plants induce the massive biosynthesis of protective proteins VSP1 and VSP2, as well as pathogenesis-related protein PR-4, which are markers of the activated jasmonate pathway. The increased contents of glutathione-S-transferases F6, F2, F10, U19, and DHAR1 and the osmotin-like defense protein OSM34 were found. The defense-associated protein PCaP1, which is required for oligogalacturonide-induced priming and immunity, was upregulated. Moreover, *rolB*-transformed plants showed the activation of all components of the PYK10 defense complex that is involved in the metabolism of glucosinolates. We hypothesized that various defense systems activated by *rolB* protect the host plant from competing phytopathogens and created an effective ecological niche for *A. rhizogenes*. A RolB → RACK1A signaling module was proposed that might exert most of the *rolB*-mediated effects on plant physiology. Our proteomics data are available via ProteomeXchange with identifier PXD037959.

## 1. Introduction

Of the *plast* family of *Agrobacterium* T-DNA oncogenes, *rolB* from *A. rhizogenes* (*Rhizobium rhizogenes*) and *6b* from *A. tumefaciens* have been studied extensively for many years [1,2]. The *rolB* oncogene of *A. rhizogenes* causes a tumor phenotype of transformed plants and perturbs the hormonal signaling pathways [3,4]. The *rolB* gene promotes de novo meristem formation in plant tissues [5,6], decreases ROS levels, and represses apoptosis [7,8]. These traits are associated with the high resistance of *rolB*-transformed cells to the ROS-inducing herbicides [7]. It was recently shown that *rolB* confers tolerance of *Arabidopsis* plants to drought stress [9]. Bettini et al. [10,11] showed the role of *rolB* in photosynthesis, demonstrating significantly increased non-photochemical quenching in *rolB*-transformed tomato plants. Wang et al. [12] suggested a role of *rolB* in lipid transport and metabolism.

New data about the influence of *rolB* on the development and morphology of plants is constantly being published. *Kalanchoë blossfeldiana* plants showed altered root system architecture depending on the strength of *rolB* expression [13] and showed a smaller plant size with fewer leaves [14]. The *Ib-rolB/C* gene isolated from naturally transgenic *Ipomoea batatas* plants induces early flowering phenotype and altered leaf morphology in transgenic *Arabidopsis thaliana* plants [15]. The involvement of the *Ib-rolB/C* gene in the control of flowering is due to its influence on the expression of genes encoding FLOWERING LOCUS T (FT), TWIN SISTER of FT (TSF), and the MADS-box protein FLOWERING LOCUS C (FLC). A mediator of the red- and far-red light signaling pathway, the *PIF4* gene (encoding phytochrome-interacting factor PIF4) was significantly overexpressed in Ib-rolB/C-transgenic lines [15], this supporting the important observation of Bettini et al. [11], which points to a role for *rolB* in far-red enriched light dissipation pathways.

Tomato plants transformed with the *rolB* gene showed a high level of resistance to fungal pathogens [16]. Some transformed lines were completely resistant to *Alternaria solani* and *Fusarium oxysporum* that are air and soilborne pathogens, respectively. The production of secondary metabolites has been also attributed to defense functions activated by 6b and RolB. It has been hypothesized that the enhancement of phytoalexin biosynthesis is associated with the suppression of competing microorganisms by agrobacteria [17,18]. Nevertheless, the defense functions of the host plant modulated by the oncogene remain largely unknown.

We previously conducted a proteomics experiment with transgenic *Arabidopsis* calli in which the *rolB* plant oncogene was constitutively expressed [19]. In particular, we noted that the abundance of the receptor for activated C kinase 1A (RACK1A) was decreased in *rolB*-transformed cells. RACK1 is a WD-40-type scaffold protein that is conserved in eukaryotes, and it plays regulatory roles in diverse signal transduction and stress response pathways. RACK1A, being a versatile protein capable of interacting directly or indirectly with many ligands, is positioned as a central hub for the integration of multiple pathways affecting key cellular functions [20,21,22]. If the RolB oncoprotein affects RACK1A abundance, this could explain the pleiotropic effects observed in transformed plants. Indeed, it is known that RACK1A is involved in immune responses [23,24] and exerts important functions in disease-resistance against fungal and bacterial phytopathogens [21,23,25,26]. 

The role of RACK1 in plant–virus interactions has been studied in the model of *Red clover necrotic mosaic virus* in tobacco [27]. This virus captures RACK1 and uses it for its own replication. If RACK1 is depleted, viral translation is impaired, which result in unsuccessful viral infection. The downregulation of *RACK1* inhibited viral translation and ROS production mediated by the p27 virus replication protein. Since ROS is essential for the infection process, downregulation of RACK1 significantly limits viral infection [27]. A similar effect of RACK1 silencing was reported for distantly related human and fly viruses [28,29]. Importantly, the inhibition of RACK1 did not affect *Drosophila* or human cell viability and proliferation [28]. *Arabidopsis rack1a* mutants were viable and less sensitive to auxin and displayed pleiotropic phenotypes resembling the phenotypes of some auxin mutants [30].

Since RACK1A is a pleiotropic regulator and is involved in modulating protection against abiotic and biotic stress, ROS metabolism, protein biosynthesis, photosynthesis, hormonal responses, developmental processes, and miRNA production, it functions at different layers of signaling pathways [21,31]. Guo et al. [31] found 215 RACK1A-interacting proteins. In total, 295 RACK1A-interacting proteins have been reported so far (https://thebiogrid.org/23627/summary/arabidopsis-thaliana/atarca.html, accessed on 15 December 2022).

Because the function of the RolB protein is not clear, we tried to determine which protective proteins are activated in *rolB*-transformed plants and to understand whether these proteins are included in the signaling network of the PACK1A regulator. It has been assumed that some defense proteins would be activated based on data on the resistance of transformed plants to phytopathogens [16]. However, it was not clear what protective signaling systems were involved. Proteomic analysis could help identify these signaling systems, or at least provide a working hypothesis for a defense mechanism.

Therefore, the aim of the present investigation was to study changes in protein levels in *Arabidopsis* transgenic plants expressing *rolB.* We performed a proteomic analysis with particular emphasis on defense proteins. Our analysis showed that *rolB* affects expression of numerous defense proteins regulated by different regulatory pathways.

## 2. Results

### 2.1. AtB-1 Line

In the present study, we used a plant clone called AtB-1, which is a cloned variant of the A4-*rolB*-transformed B5 line [9]. The phenotype of the B5 line was described in detail in a recent article by Veremeichik et al. [9] and is similar to the previously described phenotype of *Arabidopsis* plants transformed with the *rolB* gene [32]. The AtB-1 line does not show signs of necrosis or dwarfism and thus is suitable for investigating the effects of *rolB*. To quantify the copy number of *rolB* cDNA (equivalent to mRNA copy numbers) in the AtB-1 line, we used the absolute quantification method with the qPCR technique. The reaction efficiency and linearity for the serially diluted standard (PCR amplicon of *rolB*) were of good quality (Supplementary Figure S1). According to our data, 1198 ± 70 copies of *rolB* cDNA were transcribed from 1 µg of total plant RNA.

### 2.2. General Description of Proteomic Analysis

Total protein fractions from control and *rolB*-transformed *A. thaliana* plants were separated by two-dimensional gel electrophoresis. Overall, 1500 proteins were resolved on 2-D gels, and 300 of these were identified using MALDI mass spectrometry. A total of 77 overexpressed proteins and 64 underexpressed proteins were identified in the *rolB-*transformed plants. These proteins were grouped according to their function using the UniProtKB and TAIR databases. Functional groups are represented by the following proteins (the number of upregulated/downregulated proteins is indicated in brackets): chaperones (5/7), proteins involved in plant defense (21/4), response to abiotic stress (9/4), photosynthesis (2/14), response to oxidative stress and maintenance of redox homeostasis (13/0), plant development (5/0), primary metabolism (18/20), and protein biosynthesis (0/12). These proteins are presented in Table 1 and Table 2.

### 2.3. Defense Reactions and Plant Immunity

**Figure 1:**
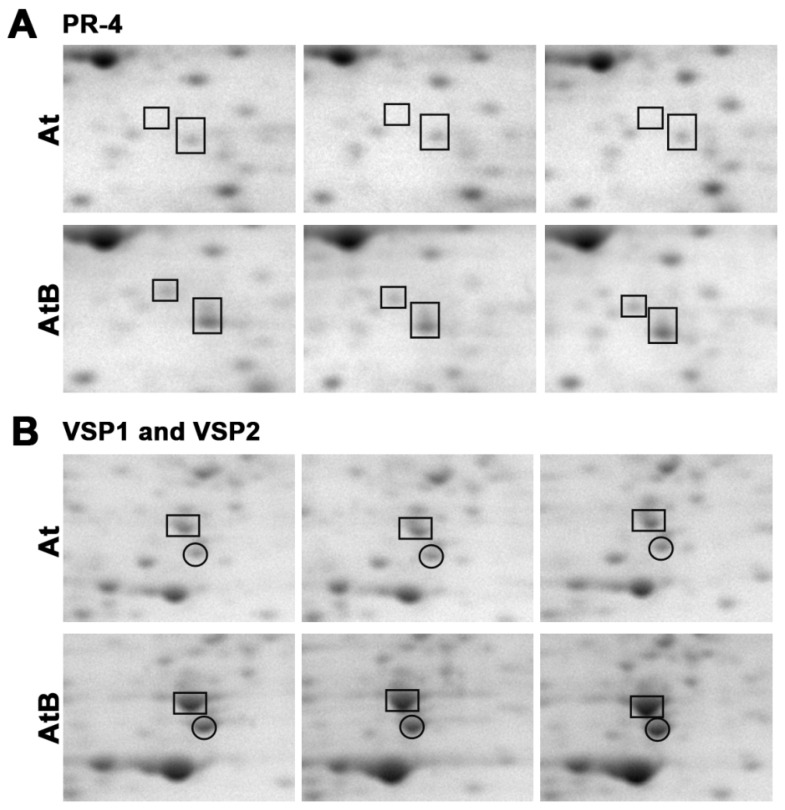
Induction of defense proteins by the *rolB* gene. Fragments of two-dimensional gels of protein fractions from the control (At) and *rolB-*transformed AtB-1 (AtB) plants obtained in three separate experiments are presented. Gels were scanned using a VersaDoc MP 4000 system with PDQuest 8.0.1 Advanced software as described in Section 4.3. Protein expression is presented as the average total spot intensity in a group of three repeated gels. (**A**) hevein-like preproprotein PR-4/HEL, marked with boxes (this protein was divided on gels into two isoforms by isoelectric mobility and by mass); (**B**) vegetative storage protein 1 (VSP1, boxes) and vegetative storage protein 2 (VSP2, circles).

We found that 21 defense-related proteins were upregulated in *rolB* plants (Table 1). A compact group of proteins constitutes the endoplasmic reticulum (ER) PYK10 defense complex. In *Arabidopsis*, the PYK10 complex forms protein aggregates inside cells consisting of β-glucosidases, GDSL lipase-like proteins, and cytosolic jacalin-related lectins [33,34]. Components of the ER PYK10 complex (such as PYK10-binding protein 1; beta-glucosidases 23 (PYK10), 37, and 18; GDSL esterase/lipase ESM1; jacalin-related lectin 23; jacalin-related lectin 35; and two defense lectin-like proteins) were upregulated (Table 1), suggesting that *rolB* is involved in plant immunity and possibly in the metabolism of glucosinolates that protect plants from pests and fungi [33,35]. Other upregulated defense proteins were represented by pathogenesis-related protein 4 (PR-4/HEL), osmotin-like protein OSM34, vegetative storage proteins 1 and 2 (VSP1 and VSP2), polygalacturonase inhibitor 1 (PGIP1), class V chitinase, avirulence-induced gene 2 protein B, and plasma membrane-associated cation-binding protein 1 (PCaP1). A representative view of relative content of some defense proteins is shown in Figure 1 and Supplementary Figure S2.

PR-4/HEL was reported to have a strong antifungal activity by crossing the pathogen hyphal membrane and interacting with a fungal fruiting body lectin [36]. OSM34 also has antifungal activity and belonged to the PR-5 family of defense proteins [37,38]. It is also involved in abiotic stress reactions via the ABA signaling pathway [39]. VSP1 and VSP2 are part of the local response of plants to herbivore attack [40]. PGIP1 limits the growth of invasive fungal pathogens and provides protection against cyst nematodes [41,42]. Some upregulated proteins involved in the response to oxidative stress and redox homeostasis also play a protective role against pathogens. These are peroxidase 34 and glutathione S-transferase F6 (Table 1).

Several defense proteins were downregulated in *rolB* plants (Table 2). These are members of RNA-binding proteins: glycine-rich RNA-binding protein 7 (GRP7) and two related RNA-binding proteins CP29B and CP31B, which are collectively involved in innate immune response [43]. Probable glucan 1,3-alpha-glucosidase, which is required for sustained activation of EFR-mediated signaling, and polyadenylate-binding protein 8 (PABP-8), involved in plant–virus interaction, were also downregulated.

### 2.4. Primary Metabolism

Changes in the primary metabolism caused by the expression of the *rolB* gene in plants have been noted previously [44]. Our data also indicate a large number of proteins of this group whose expression increased or decreased in *rolB*-transformed plants (Table 1 and Table 2). Upregulated proteins involved in carbohydrate metabolism are represented by aldose 1-epimerase, galactose mutarotase-like superfamily protein, and UDP-glucuronic acid decarboxylase 3 (Table 1). Of these proteins, aldose 1-epimerase showed a tenfold increase in abundance in the *rolB*-transformed plants. This is an interesting enzyme for our analysis, as *Phytophthora* pathogens use their own aldose 1-epimerase as a pathogenicity effector [45]. Given that fungi and bacteria do not contain genes encoding aldose 1-epimerases [45], *rolB* may create a more favorable niche for *A. rhizogenes* by activating plant aldose 1-epimerase for efficient sugar uptake. A similar function of other *plast* genes, *rolC* and *6b*, was previously proposed by Otten’s group as an ancestral property of the *plast* genes [46].

Upregulated proteins involved in lipid metabolism are represented by 3-ketoacyl-CoA thiolase 2 (KAT2), UDP-sulfoquinovose synthase, and short-chain dehydrogenase/reductase SDRA (Table 2). This data is consistent with Wang et al. [12], who proposed a role for *rolB* in lipid metabolism. Interestingly, KAT2 is involved in inflorescence development by regulating peroxisomal β-oxidation [47] and benzoylated glucosinolate biosynthesis [48], i.e., KAT2 may be involved in both processes affected by *rolB*. At the same time, several proteins involved in lipid metabolism were suppressed, such as the GDSL esterase/lipase At1g29660, acetyl-coenzyme A carboxylase carboxyl transferase, and 3-oxoacyl-[acyl-carrier-protein] synthase I (Table 2). 

Other downregulated proteins included proteins involved in amino acid biosynthesis and protein biosynthesis (Table 2), supporting the well-known inhibitory effect of *rolB* on growth. Interestingly, three proteins involved in protein degradation (ubiquitin carboxyterminal hydrolase 6, puromycin-sensitive aminopeptidase, and DNA-DAMAGE INDUCIBLE 1 protein) were inhibited, which might indicate that *rolB* simultaneously affects two oppositely directed processes in plants, such as protein biosynthesis and protein degradation.

### 2.5. Chaperones

Previously, we showed that *rolB* affects the expression of chaperone-type proteins such as heat-shock proteins (HSPs) and cyclophilins [19]. In the present investigation, we detected increased expression of heat shock 70 kDa proteins BIP1 and BIP2 (AtHsp70-11 and AtHsp70-12) in *rolB*-transformed *Arabidopsis* plants (Table 1, Supplementary Table S2). Expression of peptidyl-prolyl cis-trans isomerases CYP18-3 (ROC1), CYP19-3 (ROC2), CYP19-1 (ROC3), and CYP18-4 (ROC5) was also increased. ROC1 induces plant immunity via ROC1–RIN4 interaction and regulation of RPM1 level [49] and confers *Arabidopsis* cold tolerance by modulating jasmonic acid signaling [50]. ROC2 regulates seedling growth by affecting the expression levels of abscisic acid signaling genes [51]. ROC3 is also involved in the control of plant immunity [52].

Several proteins having chaperone function were downregulated in *rolB* plants. These are Hsp70-Hsp90 organizing protein 1 (HOP1); chaperone proteins ClpB1, ClpB3, and ClpC1; and ankyrin repeat domain-containing protein 2A (AKR2A) (Table 2). HOP proteins form large protein complexes as a part of defensome in ER, along with chitin elicitor receptor kinase 1 (CERK1), RAC1, and HSP90, thereby participating in plant antifungal defense and antiviral defense [53]. HOP proteins are also involved in plant response to cellular and environmental stresses, especially in long-term acquired thermotolerance [53]. Among downregulated proteins, we detected chaperone proteins ClpB1 and ClpB3, which are involved in establishing heat stress tolerance, and chaperone protein ClpC1, which is necessary for proper chloroplast organization. Another downregulated protein was ankyrin repeat domain-containing protein 2A (AKR2A) exhibiting chaperone activity toward chloroplast outer envelope membrane, mitochondrion outer membrane, and ER membrane. The next downregulated proteins were dehydrins ERD10 and ERD14 (Table 2). ERD10 and ERD14 are members of the dehydrin family that accumulate in response to abiotic stresses to prevent the heat-induced aggregation of various proteins [54]. 

### 2.6. Photosynthesis

There are two opposite points of view on the effect of *rolB* on the photosynthetic apparatus. Chlorophyll content was reduced in *Kalanchoë blossfeldiana* plants expressing *rolB* compared to WT plants [14]. Kodahl et al. [32] suggested that the light green color of *A. thaliana* transformed with *rolB* was caused by a decrease in chlorophyll content. On the contrary, in *rolB*-transformed tomato plants, the efficiency of photosynthesis was higher than in control plants, and the content of chlorophyll also increased and the process of non-photochemical quenching was activated [10]. This information was complemented by an observation indicating that the light energy, directed through regulated or unregulated dissipation pathways, differed between *rolB* transgenic and control plants, particularly after exposure to far-red-enriched light [11].

We have shown that ferredoxin-NADP-reductase and root isoenzyme 1/RFNR 1 are activated 2-5-fold in *rolB* plants (Table 1). This is important because ferredoxin--NADP^+^ reductases catalyze the final step in linear electron transfer (LET), and their inhibition decreases photosynthetic capacity [55]. High-light grown plants have a higher overall capacity for LET, coupled with increased resistance to photoinhibition.

However, 14 chloroplastic photosynthetic proteins were downregulated in *rolB*-transformed plants (Table 2). Five of these are involved in chlorophyll biosynthesis: magnesium protoporphyrin IX methyltransferase, coproporphyrinogen-III oxidase 1, uroporphyrinogen decarboxylase 2, protochlorophyllide reductase B, and protochlorophyllide reductase C. Leaf isozymes, such as ferredoxin–NADP reductase 1/FNR 1 and ferredoxin–NADP reductase/FNR 2, were almost two-fold downregulated. It turns out that the leaf forms of FNR are inhibited, and the root form is activated, but the physiological role of such differences is not yet clear. 

The expression of some proteins responsible for photosynthetic electron transport (ATP synthase gamma chain 1/ATPC1, plastocyanin major isoform, and cytochrome b6-f complex iron-sulfur subunit) and for chloroplast organization (protein TIC 40, signal recognition particle 54 kDa protein, and membrane-associated protein VIPP1) are also inhibited. Since the increased level of FNR 1, FNR 2, ATPC1, and VIPP1 correlates with high light intensity [55], it can be assumed that the decrease in the expression of these proteins in *rolB* plants reflects plants acclimatizing to low light intensity.

### 2.7. RACK1-Associated Proteins

**Figure 2:**
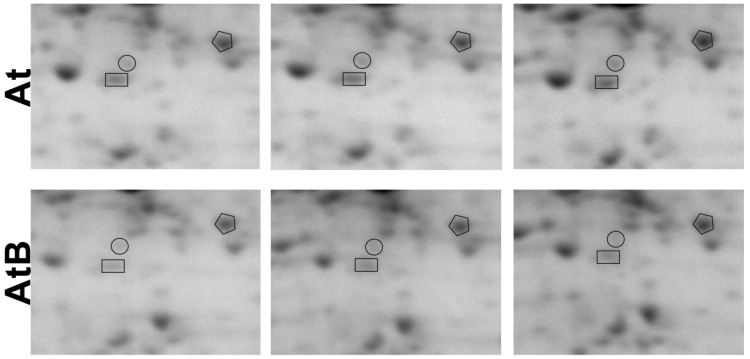
Downregulation of RACK1 proteins in *rolB*-expressing *Arabidopsis* plants. Gels were scanned using a VersaDoc MP 4000 system with PDQuest 8.0.1 Advanced software as described in Section 4.3. Protein expression is presented as the average total spot intensity in a group of three repeated gels. Fragments of 2-D gels of protein fractions from the control (At) and *rolB-*transformed AtB-1 line (AtB) are presented in triplicate: RACK1A, marked with polygons; RACK1B (boxes) and RACK1C (circles).

We tested the hypothesis that the *rolB* gene-mediated physiological effects might be manifested through RACK1 proteins. If RACK1A, RACK1B, and RACK1C are suppressed in *Arabidopsis* plants, it would result in various changes in physiological functions. Indeed, the abundance of RACK1A, RACK1B, and RACK1C was decreased in the *rolB*-transformed plants, as shown in Figure 2 and Table 1. RACK1A expression was suppressed 1.5-fold both in *rolB*-transformed plants (Table 2) and in calluses [19] and was inhibited almost 4-fold for RACK1B and RACK1C (Table 1). 

The BioGRID annotation (https://thebiogrid.org/23627/summary/arabidopsis-thaliana/atarca.html, accessed on 15 December 2022) shows that the most abundant groups of RACK1A-interacting proteins are represented by ROS-detoxifying enzymes, ribosomal proteins, and photosynthetic proteins.

It is known that RACK1A physically interacts with numerous ROS-detoxifying proteins such as APX1, CAT3, CSD1, FCD1, DHAR3, GPX1, and others (BioGRID annotation). In our analysis, we detected proteins of this group, including peroxidases, peroxiredoxins, glutathione transferases, superoxide dismutases, ascorbate peroxidase 1, and others that are listed in Table 1. All of these were upregulated in the *rolB*-transformed plants.

A large group of proteins related to protein biosynthesis also interacts with RACK1A. They include 20S, 40S, 50S, and 60S ribosomal proteins (BioGRID). We found that 30S ribosomal protein S1, 40S ribosomal protein S3-1, 40S ribosomal protein S3-3, 50S ribosomal protein L4, 50S ribosomal protein L12-1, 50S ribosomal protein L1, 60S ribosomal protein L5-1, and 60S ribosomal protein L5-2 were downregulated in the *rolB*-transformed plants (Table 2).

The third large group of RACK1A-interacting proteins is represented by proteins of the photosynthetic apparatus such as CAB1, CAB3, LHCA1, LHCA2, LHCA3, APE1, and others (BioGRID). Among the upregulated proteins in the *rolB*-transformed plants were FNR 1 (ferredoxin-NADP reductase, root isozyme 1) and bifunctional protein FolD2 (tetrahydrofolate dehydrogenase/cyclohydrolase 2). Several photosynthetic proteins were downregulated (Table 2). 

Table 3 presents RACK1A-interacting proteins whose abundances were changed in AtB-1 plants. The expression of 11 RACK1A-interacting proteins was increased in *rolB* plants while expression of four proteins was decreased. Most (6/11) of the upregulated proteins are related to plant defense (Table 3). These results suggest that some of the processes affected by the *rolB* gene, such as antioxidant defense [7], plant growth suppression [14,32], and changes in the photosynthetic process [10,11], may be associated with changes in the content of RACK1 proteins.

### 2.8. CERK1-Associated Proteins

Being involved in defense reactions against plant pathogens, RACK1A acts as an adaptor in the formation of complexes with heterotrimeric G proteins, linking it to the MAPK cascade in the PrpL/ArgC-triggered immune signaling pathway [23,24]. Arabidopsis G proteins have also been implicated in plant immunity mediated by multiple receptor-like kinases (RLKs) including flagellin sensing 2 (FLS2), receptor-like kinase EFR, and chitin elicitor receptor kinase 1 CERK1, which recognize bacterial flagellin, elongation factor-Tu (EF-Tu), and fungal chitin, respectively [66]. Using the BioGRID, IntAct, and STRING databases, we searched for FLS2-, EFR-, and CERK1-interacting partners that were detected in our analysis. We found that the *Arabidopsis rolB* interactome is enriched with proteins physically interacting with CERK1 but not with FLS2 or EFR. The CERK1-interacting proteins were represented by annexin D1, superoxide dismutase [Cu-Zn] 2, vegetative storage protein 2, beta-glucosidase 1 (AtBG1), L-ascorbate peroxidase 1 (Table 1), and RGG repeats nuclear RNA binding protein C and ferredoxin-NADP reductase FNR 2 (Table 2). 

### 2.9. Gene Expression

**Figure 3:**
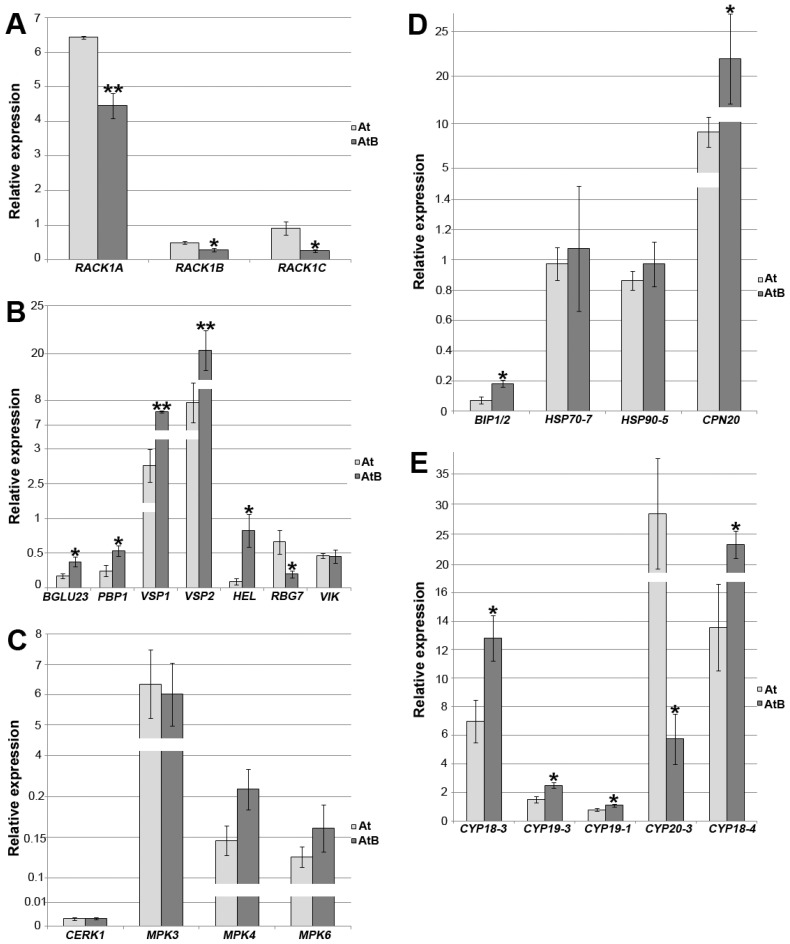
qPCR analysis of selected genes in *Arabidopsis* normal (At) and *rolB*-transformed AtB-1 line (AtB). (**A**) expression of *RACK1* genes; (**B**) expression of genes involved in defense reactions and plant immunity; (**C**) CERK1-associated genes; (**D**,**E**) chaperone-type genes. Data are presented as mean relative expression fold ± standard error across three different experiments with three technical replicates. Asterisks above the bars indicate significantly different mean values (* *p* < 0.05; ** *p* < 0.01), Fisher’s LSD.

To confirm the results of the proteomic analysis, we performed qPCR analysis with individual genes corresponding to the most interesting proteins whose abundances changed. As expected, the expression of the *RACK1A*, *RACK1B,* and *RACK1C* genes was reduced in *rolB*-transformed plants (Figure 3A, Supplementary Table S2). In accordance with the proteomic data, the expression of genes involved in defense reactions and plant immunity, such as *PR-4/HEL*, *VSP1*, and *VSP2*, as well as encoding components of the ER-PYK10 defense complex (*PBP1* and *BGLU23/PYK10*), increased (Figure 3B). The downregulation of *RBG7* encoding GRP7 was also consistent with the proteomic data (Figure 3B, Table 2). In line with the proteomic data, the expression of genes corresponding to peptidyl-prolyl cis-trans isomerases *ROC1* (CYP18-3), *ROC2* (CYP19-3), *ROC3* (CYP19-1) and *ROC5* (CYP18-4) was increased in transgenic *Arabidopsis* plants (Figure 3E, Supplementary Table S2).

The hypothesis that CERK1-associated genes are activated in *rolB* plants was tested. The qPCR analysis showed that the expression of the *CERK1* gene is not activated in the *rolB*-transformed plants (Figure 3C). Because the connection between the CERK1-based receptor complexes with the canonical MAPK cascade is well established [67], and because the activation of the MAPK cascade is essential for plant immunity, the expression of several MAP kinase genes was studied. There were no significant differences in expression levels of the *MPK3*, *MPK4*, and *MPK6* genes in the WT and *rolB-*transformed plants (Figure 3C). Therefore, the hypothesis of activation of the CERK1-mediated pathway by the *rolB* gene and concomitant activation of MAP kinase cascades was not confirmed at the level of gene expression.

It was previously suggested that *rolB*-transformed cells activate chaperone proteins as a cell response to a biochemical imbalance caused by oncogene invasion [19]. Heat-shock 70-kDa proteins 6 and 7 (Hsp70-6 and Hsp70-7), Hsp90-5, 20-kDa chaperonin (Cpn10), and chaperonin 60 subunit α1 were activated in *rolB*-expressing *Arabidopsis* cells [19]. Such Hsp70 proteins as AtHsp70-10, AtHsp70-14, and AtHsp70-15 were in equal abundance in control and *rolB*-expressing calli [19]. Here, we showed the enhanced expression of AtHsp70-11 and AtHsp70-12 (also known as BIP1 and BIP2, Table 1), and the qPCR analysis confirmed the increased expression of the corresponding genes in the *rolB-*transformed plants. The present analysis showed that expression of selected chaperone genes did not completely coincide with that in *rolB*-calli. Although *Cpn10* was activated in the *rolB*-transformed plants as in *rolB*-calli, no significant differences were observed in the expression levels of the *Hsp70-7* and *Hsp90-5* genes between control and *rolB* plants while these genes were upregulated in *rolB*-expressing calli. This information confirmed the involvement of heat-shock proteins in the functioning of the *rolB* gene. At the same time, various forms are induced differently in plants and calluses.

A significant role of CYP20-3 (ROC4) in *rolB* effects has been proposed [19]. CYP20-3 connects redox and light signals to cysteine biosynthesis and stress responses in chloroplasts [68] and is known to be a key effector protein that links hormone signaling to redox homeostasis during stress responses. CYP20-3 interacts with the 26S proteasome subunit RPT2A and RACK1A. CYP20-3 was not found in the present analysis, but we were interested in analysis of its gene expression to compare results on calli and plants. As in *rolB*-calli, mRNA levels of *CYP20-3* were five times lower in *rolB-*transformed plants (Figure 3E, Supplementary Table S2).

The VH1-interacting kinase (VIK) was also not found in our 2D gels. The expression of the gene encoding this interesting protein (VIK is involved in the defense response to fungi, the negative regulation of programmed cell death, regulation of the plant-type hypersensitive response, and responses water deprivation; TAIR annotation) was studied. In contrast to *rolB*-calli, VIK expression was not changed *rolB-*plants, which weakens interest in this line of further research. 

In general, all data on gene expression were consistent with the proteomic analysis. Some selected genes of interest were expressed differently in *rolB*-transformed calluses and plants, probably indicating their dependence on the level of cell differentiation.

## 3. Discussion

### 3.1. Defense Response

We found an unexpectedly large number of defense-related proteins whose expression was increased in *rolB*-transformed *Arabidopsis* plants (Table 1). Previously, it was noted that some effects of *rolB* on plant physiology can be realized through the primary effects of the oncogene, as well as by triggering several layers of the host’s immune compensatory reactions [19]. From the side of the pathogen, suppression of the plant immune responses is usually observed for successful colonization. Plants adapt to invasion by activating defense mechanisms. On the other hand, a pathogen can use the plant’s defenses to its advantage to limit the spread of competing microorganisms and maintain its own ecological niche. The strategy of *A. rhizogenes* to suppress the plant immune system appears to be essential for the pathogen in the early stages of plant colonization. However, then, after the integration of T-DNA into the host genome, including the integration of the *rolB* oncogene, it becomes possible to manipulate plant signaling systems for the benefit of the *Agrobacterium*. 

The type III effector HopU1 of *Pseudomonas syringae* can suppress plant innate immunity by interacting with RNA-binding proteins GRP7, CP29B, and CP31B [69]. HopU1-induced ADP ribosylation of these RNA-binding proteins suppresses host immunity by affecting RNA metabolism and the plant defense transcriptome. There is an analogy between the effects mediated by RolB and some type III proteins of *P. syringae*. The effector HopAO1 (HopPtoD2) protein of *P. syringae* is injected from bacterial cells into plant cells to promote bacterial growth by the suppression of the innate immunity of the host cells. Interestingly, type-III effectors simultaneously activate pathways associated with stress resistance [70]. It was shown that both RolB and HopAO1 possess protein tyrosine phosphatase activity [71,72,73], suppress induced ROS in plants [7,73], and suppress induced programmed cell death [8,72]. Because GRP7, CP29B, and CP31B proteins were downregulated (Table 2), we suggest that immunity of *rolB*-expressing plants might be inhibited in this way. A member of the RolB family, the 6b oncoprotein encoded by the *6b* gene from *A. tumefaciens*, also displays ADP ribosylation activity in the presence of plant co-factors [74]. ADP ribosylation of GRP7 by the pathogen effector HopU1 is important for pathogen invasion because pathogens must block or avoid pattern-triggered immunity (PTI) to cause disease. GRP7 plays a critical role in plant immunity since the abundance and activity of GRP7 are required for establishing optimal PTI response [43]. Therefore, decreased level of GRP7 and related CP29B and CP31B proteins suggests that normal PTI response in *Arabidopsis rolB*-expressing plants might be disturbed. 

A decrease in the expression of RACK1 proteins (Table 2) in *rolB* plants might indicate a suppression of the immune status of plants since the formation of immune complexes consisting of the RACK1A, accessory proteins, and the MAPK cascade might be disrupted [23,24,25,75]. Among proteins identified in the proteomic analysis, we selected proteins that physically interact with RACK1A (using BioGRID database and data of Islas-Flores et al. [21]). We found that expression of 11 RACK1A-interacting proteins was increased in *rolB* plants while the expression of four proteins was decreased (Table 3). Surprisingly, most (6/11) of the upregulated proteins were related to plant defense (Table 3). This means that a decrease in the level of RACK1A in *rolB* plants could not lead to a weakening of plant defense responses. Consistent with this suggestion, it has been reported that during the interaction of tobacco with *Red clover necrotic mosaic virus*, RACK1 suppression inhibits translation of the virus [27]. 

Some defense systems are activated in *rolB* plants. Our analysis presents major components of the ER-PYK10 defense complex, such as PYK10 binding protein 1/PBP1, beta-glucosidases 18 (AtBG1), 23 (PYK10) and 37, GDSL esterase/lipase ESM1, jacalin-related lectin 23, jacalin-related lectin 35, and other protective lectin-like proteins. All of these proteins were upregulated (Table 1). Since ER bodies in Brassicaceae plants are important for defense against pests, nematodes, and various microbes, including fungal pathogens, at least in part through degradation products of glucosinolates [33,34,35], these results imply that *rolB* can activate the ER-body defense responses. In addition, the expression of nitrile-specifier protein 5/NSP5, which converts glucosinolates to their corresponding simple nitriles via the myrosinase pathway, was also upregulated. An independent level of defense is represented by indole glucosinolates, which play an important role in plant immunity and are biosynthesized through regulation by transcription factors MYB and MYC. In fact, increased levels of indole glucosinolates have been found in *Arabidopsis* cells expressing *rolB* [76]. 

On 2-D gels of *rolB*-plant protein fractions, we noted the appearance of massive protein spots (Figure 1) corresponding to three proteins: vegetative storage proteins 1 and 2 (VSP1 and VSP2), which are acid phosphatases and hevein-like preproprotein HEL (synonym: pathogenesis-related 4, PR-4). VSP1 is a MeJA-inducible wound-responsive protein that is involved in the local response of plants to herbivore attacks [40]. VSP2 possesses strong anti-insect activity [77] and acts via the jasmonate pathway to inhibit *Pseudomonas syringae* pv. *tomato* DC3000 during plant-pathogen interaction [78,79]. PR-4 binds chitin and is involved in defense response to fungus and herbivores [80,81]. The genes encoding these three proteins are markers of the jasmonate pathway. Therefore, the jasmonate signaling pathway might be activated in *rolB*-expressing plants.

The plasma membrane-associated cation-binding protein 1/PCaP1 is required for oligogalacturonide and flagellin-induced priming and immunity [82] and negatively regulates intercellular movement of *Bamboo mosaic virus* [83]. Its increased expression in *rolB* plants (Table 1) indicates a preformed immune response. Another upregulated protein is nitrilase NIT2, which is essential for plant defense and R-gene mediated resistance response against *P. syringae* pv. *tomato* [84].

Among classical phytopathogenic effectors, such as receptor-like kinases FLS2, EFR and CERK1, we found changes in expression of CERK1-interacting proteins. Recent data indicate that *Arabidopsis* CERK1 plays multifaceted roles beyond chitin signaling and can mediate the crosstalk between chitin signaling and other biotic or abiotic stress signaling [85,86]. In addition to the VSP2-CERK1 interaction, we found several other proteins that physically interact with CERK1 (BioGRID annotation; see Section 2.8). The expression of five of these was increased, and the expression of two was decreased. Indeed, a bioinformatics analysis showed that CERK1-interacting proteins detected in our proteomic experiment (annexin D1, superoxide dismutase [Cu-Zn] 2, vegetative storage protein 2, beta-glucosidase 1 (AtBG1), L-ascorbate peroxidase 1, RGG repeats nuclear RNA binding protein C, and ferredoxin-NADP reductase) are involved in chitin signaling, as well as biotic or abiotic stress signaling, indicating that a part of *rolB*-mediated effects might be realized via the CERK1 signaling pathway. However, neither the *CERK1* gene itself nor the genes encoding related MAP kinases (MPK3, MPK4, and MPK6; Figure 3) are activated in *rolB* plants. It seems likely that the CERK1-based receptor complex with the MAPK cascade [67] does not function in *rolB*-transformed plants.

A proteomic analysis of *A. thaliana* performed by Mukherjee et al. [80] showed a high induction of glutathione S-transferases, PR-4, and osmotin in plants infected with fungal pathogen *Alternaria brassicicola*. In our analysis, we also showed increase in abundance of glutathione S-transferases F6, F2, F10, U19, and DHAR1, PR-4 and the osmotin-like protein OSM34 (Table 1). With this data in mind, it is now possible to explain why *rolB* tomato plants were resistant to *A. solani* [16]. At the same time, we confirmed the data that the pool of antioxidant enzymes is activated in *rolB* plants, which gives a high resistance to ROS-inducing herbicides [7]. Having constantly activated defense and antioxidant systems, *rolB* plants rearrange their metabolism with concomitant growth inhibition. These processes are accompanied by an apparently repressed protein biosynthesis system because many ribosomal proteins and elongation factors are downregulated in *rolB* plants (Table 2).

The influence of *rolB* on chaperone proteins was discussed earlier [19]. Chaperone-type proteins were also identified in this work, of which the heat shock 70 kDa proteins BIP1 and BIP2 (AtHsp70-11 and AtHsp70-12), peptidyl-prolyl cis-trans isomerases CYP18-3 (ROC1), CYP19-3 (ROC2), CYP19-1 (ROC3), and CYP18-4 (ROC5) were upregulated. ROC1 plays an important role in plant defense, acting both in PTI and effector-triggered immunity (ETI) as a modifier of RIN4 (RPM1-interacting protein 4) configuration and regulator of RPM1 (resistance to *P. syringae* pv. *maculicola* 1) and RPS2 (resistance to *P. syringae* 2) proteins [49]. One significant difference was found between proteomic analyses of the *rolB* calli [19] and *rolB* plants (this study): cyclophyllin amounts were decreased in calli and increased in plants. It is not yet clear where this difference comes from, but it has been confirmed at the level of protein and gene expression. It is known that cyclophyllins inhibit *Agrobacterium*-mediated transformation in both tobacco and *Arabidopsis* [87]. The significance of this relationship in vivo is presently unknown, although it can be assumed that cyclophyllins play a role not only in the transfer of T-DNA [87], but also in a stably transformed plant. The mode of action of increased (or decreased) levels of ROC1 on the immune system affected by the RIN4-RPM1-RPS2 module depends on the type of pathogen-employed effectors [49]. Therefore, it remains to be seen how an increase in the expression of ROC1 affects immunity of *rolB*-transformed plants. Interestingly, ROC1 and PYK10-binding protein 1 are both RIN4-associated proteins [49]. The expression of both proteins is increased in *rolB* plants. The relationship between the ER defense complex PYK10 and RIN4-based module is currently unknown [49]. Similarly, the current state of relatively low coverage of protein–protein interactions in the *Arabidopsis* interactome does not allow establishing a connection between signaling systems based on RACK1 and CEPK1. So far, only one protein is known, the serine/threonine-protein kinase WNK8, which can regulate both systems (https://thebiogrid.org/19454/summary/arabidopsis-thaliana/wnk8.html, accessed on 15 December 2022).

Therefore, it is still not possible to combine the described multiple interactions into a single signaling network. Most likely, the RolB protein has multiple targets, similar to the 6b protein encoded by *6b* gene from *A. tumefaciens* [74]. In turn, these multiple targets may represent pleiotropic regulators that trigger cascades of protein interactions. RACK1A, in addition to the defense function, has other functions, such as interaction with the microRNA processing machinery, through the SERRATE (SE) and Argonaute (AGO1) proteins [88]. The involvement of *rolB* in the modulation of *SE* and *AGO1* expression was previously shown [89], and specific interactions of 6b protein with SE and AGO1 were also reported [74].

### 3.2. ROS Metabolism and Abiotic Stress Response

It has been shown that important ROS generators, NADPH oxidase genes *AtRbohD* and *AtRbohF* and corresponding proteins, are upregulated in *rolB*-expressing cells [90], and the deletion of *rolB* from wild-type hairy roots decreases ROS level in *rolB*-deficient hairy roots [12]. This data could indicate that ROS levels in *rolB*-transformed plants should be increased. However, according to previous data indicating decreased ROS levels and increased expression of genes encoding ROS-detoxifying enzymes in *rolB*-transformed cells [7], we observed the activation of ROS-detoxifying enzymes (peroxiredoxin-2B, peroxiredoxin-2F, glutathione S-transferase DHAR1, L-ascorbate peroxidase 1, superoxide dismutase [Cu-Zn] 2, superoxide dismutase [Fe] 1, and peroxidase 34/PRX34) in this work (Table 1). We found a strong, more than seven-fold, activation of expression of the apoplastic class III peroxidase 34 (PRX34) in *rolB* plants (Table 1). This result is consistent with previously published data that *rolB* induces the expression of class-III peroxidases [91]. PRX34 is an important player in ROS generation via apoplastic oxidative burst during plant–pathogen interactions, along with AtRbohD and AtRbohF, and PRX34 is positioned as major component of PTI [92,93].

Recent studies indicate that *rolB* confers drought tolerance in transgenic *Arabidopsis* plants and increases flavonoid biosynthesis [9]. These traits are associated with a change in ROS metabolism, which includes two opposite processes, such as *rolB*-dependent activation of ROS production [12,90] and ROS detoxification [7,19] and present study, which together result in ROS homeostasis under stress conditions. Drought tolerance of *rolB*-transformed plants is in accordance with the proteomics data and can be explained by the upregulation of many proteins involved in the response to water deprivation. These are plasma membrane-associated cation-binding protein 1/PCaP1, annexin D1, NADPH-dependent aldo-keto reductase, aldo-keto reductase family 4 member C8, glutathione S-transferase F10, glutathione S-transferase F6, and glutathione S-transferase U19 (Table 1). Thus, we observe two oppositely directed processes in *rolB*-transformed cells; the generation of ROS by activating NADPH oxidases and peroxidase PRX34 and ROS scavenging by activating antioxidant enzymes and flavonoid biosynthesis. The resulting effect is the establishment of the intracellular ROS level of about 60% of normal level and the stabilization of the redox balance, as revealed by stabilization of the reduced glutathione/oxidized glutathione (GSH/GSSG) ratio in *rolB*-expressing cells [7]. This situation resembles the process known as ROS-mediated acclimation of plants to stress combinations [94]. Therefore, cells expressing *rolB* are better prepared for both biotic and abiotic stresses, albeit at the expense of a trade-off between defense processes and growth. 

## 4. Materials and Methods

### 4.1. Plant Material

Wild-type (WT) *Arabidopsis thaliana* (Columbia-0) plants were obtained from seeds purchased from RIKEN BioResource Research Center (Ibaraki, Japan). *RolB*-transgenic plants were established with the floral dip method as previously described [9,95]. Plants were transformed with *A. tumefaciens* strain GV3101 containing the pPCV002-CaMVBT construct (*rolB* under the control of 35S CaMV promoter) [96]. WT and *rolB* plants were cultured on half strength hormone-free W_0_ solid medium with 16 h/8 h light/dark cycles (24 °C/120 µmol m^−2^ s^−1^; light source: warm white LED lamps with emission maxima at 581 nm and 448 nm) for three weeks. The W_0_ medium contained standard Murashige and Skoog macrosalts, microsalts, and Fe-EDTA, with the exception of NH_4_NO_3_, whose concentration was decreased to 400 mg/L. The following components were added to W_0_ medium (mg/L): thiamine-HCl (0.2), nicotinic acid (0.5), pyridoxine-HCl (0.5), meso-inositol (100), peptone (100), sucrose (10,000), and agar (6000). All reagents were obtained from Sigma-Aldrich (St. Louis, MO, USA, “Tissue Culture Grade”).

In the present study, we used a plant clone called AtB-1 derived from A4-*rolB*-transformed line B5 [9]. Briefly, the seeds of antibiotic resistant F1 plants were germinated in the presence of kanamycin. Well growing kanamycin resistant F2 plants were analyzed to determine the transfer and expression of A4-*rolB*. Based on molecular analysis and morphological features characteristic of transformed plants (dwarfing and abnormal flowering), clone B5 was selected. From the second generation F2, the F3 generation of B5 line was selected to obtain plants for proteomic analysis. The F3 generation of B5 line exhibited a faster transition into the reproductive phase and earlier flowering compared to WT plants, but it did not show signs of dwarfism [9]. Real-time PCR analysis showed that the haploid B5 genome carries a single copy of the integrated T-DNA [9]. Next, the B5 line growing in soil was transferred to an in vitro culture (to avoid microbial contamination, which could distort the results of proteomic analysis) under the name AtB-1.

AtB-1 represented a moderately expressing *rolB* line, in which the oncogene was expressed at the level of 1200 copies of *rolB* cDNA from 1 µg of the total plant RNA. No signs of cell death or necrosis were observed in AtB-1 plants; *rolB*-transformed and WT plants grew well and demonstrated normal green color.

### 4.2. 2D-Gel Electrophoresis

Reagents were purchased from Sigma-Aldrich (St. Louis, MO, USA), unless otherwise noted. Proteins were isolated from the aerial parts of WT and AtB-1 A. thaliana plants (1 g fresh weight) using a phenol extraction methanol/ammonium acetate precipitation method as described [19]. A protein from each extraction type was quantified using Bradford assay. For isoelectric focusing, dried protein pellets were dissolved in IPG buffer, containing 9.5 M urea with thiourea, 4% *w*/*v* CHAPS, 65 mM DTT, 2% Pharmalyte pH 3-10 (GE Healthcare, Uppsala, Sweden), and 0.01% *w*/ bromophenol blue. Protein probe diluted in IPG buffer was loaded to 18-cm Immobiline DryStrip pH 3–10 NL (GE Healthcare, Uppsala, Sweden) according to the manufacturer’s recommendations by passive rehydration for 12 h at 20 °C. IEF was performed in a Protean IEF Cell (Bio-Rad Laboratories Inc., Hercules, CA, USA) for 60,000 V-h as described [19]. For SDS-PAGE, 12% polyacrylamide gels with 4% stacking gels were run in a Protean II xi cell (Bio-Rad Laboratories Inc., Hercules, CA, USA). The gels were stained with Coomassie Brilliant Blue G-250. A set of three control and three experimental gels was used in the analysis.

### 4.3. Quantification of Protein Expression

Gels were scanned using the VersaDoc MP 4000 System (Bio-Rad Laboratories Inc., Hercules, CA, USA). PDQuest 8.0.1 Advanced software (Bio-Rad Laboratories Inc., Hercules, CA, USA) was used for the analysis of the protein maps. The Spot Detection Wizard was used to select the parameters for spot detection, such as a faint spot and a large spot cluster. The results of automated spot detection were checked and manually corrected. A local regression model (Loess) was used for normalization of spot intensity. The protein expression was accessed using PDQuest 8.0.1 Advanced software and was presented as mean total intensity of a defined spot in a replicate gel group. Spot quantity is the sum of the intensities of pixels inside the boundary. The fold of protein expression change was accessed based on mean protein intensity. For quantitative differentiation, a 1.5-fold change or higher in the average spot intensity was regarded as significant. Statistical significance of differences was assessed using Student’s *t*-test at a significance level of 0.05 in three replicates. 

### 4.4. Experimental Design and Statistical Rationale

Three biological experiments were carried out with three technical replicates. The total number of samples analyzed by MALDI was 300. The number of technical replicates for protein identification by MALDI mass spectrometry was 2-3 (up to 5 for important and low-abundance proteins). Individual protein spots, selected on the basis of image-analysis output, were excised and digested in-gel with trypsin (Trypsin V511, Promega, Madison, WI, USA) as previously described [19]. For MALDI-TOF identification, 0.5–1 μL of the sample (50% solution of acetonitrile in water, 0.1% TFA) was placed on a ground steel MALDI target plate or AnchorChip or SmallAnchor (depending on the protein quantity), and 0.5–1 μL of the matrix (α-cyano-4-hydroxycinnamic acid) (Bruker Daltonics, Bremen, Germany) was added. 

### 4.5. MALDI-TOF Mass Spectrometry and Protein Identification

All mass spectra were acquired with an Autoflex (Bruker Daltonics, Bremen, Germany) MALDI-TOF mass spectrometer with a nitrogen laser operated in the positive reflector mode (standard method RP 700-3500 Da.par) under the control of FlexControl software (version 3.4; Bruker Daltonics, Bremen, Germany). The analysis was performed in the automatic mode (AutoXecute—automatic Run). The spectra were externally calibrated using the CalibratePeptideStandards. FAMSMethod, and a standard calibration mixture (Protein Calibration Standard I, Bruker Daltonics, Bremen, Germany). The data files were transferred to Flexanalysis software version 3.4 (Bruker Daltonics, Bremen, Germany) for automated peak extraction. Assignment of the first monoisotopic signals in the spectra was performed automatically using the signal detection algorithm SNAP (Bruker Daltonics, Bremen, Germany). For MS and MS/MS analyses, we used the PMF.FAMSMethod and SNAP_full_process. FALIFTMethod, respectively. Each spectrum was obtained by averaging 1500–5000 laser shots (300 shots in a step) acquired at the minimum laser power. The data was analyzed using BioTools (version 3.2; Bruker Daltonics, Bremen, Germany). A peptide mass tolerance of 0.5 Da and a fragment mass tolerance of 0.5 Da were adopted for database searches. The *m*/*z* spectra were searched against the *Arabidopsis thaliana* NCBInr and SwissProt databases using the Mascot search engine. Threshold score was 40. Further data were analyzed using UniProtKB (http://www.uniprot.org/uniprot/, accessed on 12 June 2022) and other specialized databases and programs. The mass spectrometry proteomics data has been deposited to the ProteomeXchange Consortium via the PRIDE [97] partner repository with the dataset identifier PXD037959 and 10.6019/PXD037959.

### 4.6. RNA Isolation, cDNA Synthesis, and Real-Time PCR

#### 4.6.1. RNA Isolation and cDNA Synthesis 

RNA samples were isolated from the aerial parts of WT and AtB-1 plants cultivated in vitro for 21–24 days. The experiments were carried out in triplicate. RNA concentration, purity, and integrity were estimated with OD measurement via BioSpec-nano (Shimadzu Europa GmbH, Duisburg, Germany) and non-denaturing agarose gel electrophoresis. The total RNA (1 μg) was reverse transcribed using M-MuLV–RH Kit (Biolabmix, Novosibirsk, Russia) with the oligo(dT)_20_ primer. The cDNA produced was then diluted 5-fold with nuclease-free water and stored at −20 °C for subsequent analysis. The reaction with all ingredients, including the same amount of mRNA (1 μg), except reverse transcriptase, was used as a control without reverse transcriptase 

#### 4.6.2. Quantitative Real-Time PCR

Quantitative real-time PCR (qPCR) analysis was performed using a CFX96 (Bio-Rad Laboratories, Hercules, CA, USA) with 5× qPCRmix-HS SYBR master mix (Evrogen, Moscow, Russia) following the manufacturer’s recommendations. Three biological replicates, resulting from three different RNA extractions, were used for analysis. All samples, including the external standards, no reverse transcriptase control, and no template control, were run in triplicate. *A*. *thaliana RHIP1* was used as a reference gene [98,99]. The primer sets used in the analysis are listed in Supplementary Table S1. Data was analyzed using CFX Manager Software (Bio-Rad Laboratories Inc., Hercules, CA, USA). 

#### 4.6.3. Absolute Quantification

Absolute RT-PCR links the PCR signal to the entered copy numbers using a calibration curve. PCR amplicon of *rolB* was used as a standard. PCR amplicon of 780 bp was amplified from *A. tumefaciens* GV3101 strain carrying pPCV002-CaMVBT construct by PCR with gene-specific primers *rolB_780-D* and *rolB_780-R* [10] (Supplementary Table S1). The product was then separated by agarose gel electrophoresis followed by purification using the Cleanup Standard DNA Purification Kit (Evrogen, Moscow, Russia). The concentration of the purified DNA standard was determined with a BioSpec-nano (Shimadzu, Europa GmbH, Duisburg, Germany) and converted to number of copies per microliter using its molecular weight and Avogadro constant according to [100]. The standard was finally prepared for qPCR in 10-fold dilution series, ranging from 1.25 × 10^1^ to 1.25 × 10^5^ copies per 1 µL. A calibration curve was generated using CFX Manager software (Bio-Rad Laboratories, Hercules, CA, USA) by plotting the quantitative cycle (Cq) corresponding to each standard dilution as a function of the value of its corresponding logarithmic concentration number (expressed in copies per 1 µL).

#### 4.6.4. Statistical Analyses

The statistical analysis was performed using Statistica 10.0 (StatSoft Inc., Tulsa, OK, USA), with the level of statistical significance being regarded as *p* < 0.05. Two independent categories were compared using the Student’s *t* test while comparisons among multiple groups were performed using analysis of variance (ANOVA), followed by a multiple comparison protocol. The inter-group comparison was made using Fisher’s protected least significant difference (PLSD) post-hoc test.

## 5. Conclusions

Tyrosine (de)phosphorylating activity is a prerequisite for the proper activity of RACK1 in animal models [101], where RACK1 deregulation is often associated with tumor progression. Likewise, tyrosine phosphorylation regulates *Arabidopsis* RACK1A activity [102]. Since RolB has been shown to have tyrosine phosphatase activity (https://www.uniprot.org/uniprot/P20402, accessed on 3 April 2022 [71]), we suggest that RACK1A is one of the potential targets for the RolB protein. The RolB → RACK1A signaling chain may be sufficient for physiological effects mediated by RACK1A, such as modulation of abiotic and biotic stress defense, ROS metabolism, protein biosynthesis, photosynthesis, hormonal responses, developmental processes, and miRNA production. This scenario seems realistic, since all these features are inherent in *rolB*-transformed plants and are described in the literature and in this study.

Summarizing the results of the proteomic analysis, we conclude that *rolB* causes changes in the functioning of various defense systems in transformed *Arabidopsis* plants. The primary effect of the oncogene aimed at suppressing the plant’s immune system appears to include suppression of PTI, by analogy with the action of bacterial type-III effectors, such as HopU1 of *P. syringae*. However, after the successful invasion and formation of tumor tissue, the *rolB* oncogene induces a defense effect through various pathways. The first of these is the suppression of RACK1A, RACK1B, and RACK1C proteins, similar to the effect of viral translation inhibition caused by the suppression of RACK1 when tobacco interacts with *Red clover necrotic mosaic virus* [27]. Simultaneously, the *rolB* gene induces the biosynthesis of glucosinolates [76], which are defense compounds against pests and fungi. We then showed that plants transformed with the *rolB* gene induce massive biosynthesis of jasmonate-induced defense proteins VSP1, VSP2, and PR-4 to protect plants from fungi and herbivores. The induction of PGIP-1 and other related proteins is an important factor in resistance to phytopathogenic fungi. In addition, *rolB* plants activate components of the PYK10 ER defense complex involved in the metabolism of glucosinolates. Therefore, we believe that most *rolB*-activated defense systems are aimed at protecting the host from competing phytopathogens and creating an effective ecological niche for *A. rhizogenes*.

## Figures and Tables

**Table 1:** Proteins upregulated in *rolB*-expressing *Arabidopsis* plants.

	UniProtKB Code	Name of the Protein/Short Name	Protein Function ^1^	Activation, Folds ^2^
**Chaperones**				
1	Q38867	Peptidyl-prolyl cis-trans isomerase CYP19-3/ROC2	Protein peptidyl-prolyl isomerization	3.8
2	P34790	Peptidyl-prolyl cis-trans isomerase CYP18-3/ROC1	Protein peptidyl-prolyl isomerization	2.0
3	Q42406	Peptidyl-prolyl cis-trans isomerase CYP18-4/ROC5	Protein peptidyl-prolyl isomerization	1.7
4	Q38900	Peptidyl-prolyl cis-trans isomerase CYP19-1/ROC3	Protein peptidyl-prolyl isomerization	1.5
5	Q9LKR3 Q39043	Heat shock 70 kDa protein BIP1/AtHsp70-11 Heat shock 70 kDa protein BIP2/AtHsp70-12	Protein refolding; required for pollen development and pollen tube growth	2.8
**Plant defense**				
1	P43082	Hevein-like preproprotein/PR-4/ HEL	Defense response to bacterium, fungus, and virus; systemic acquired resistance	10
2	P50700	Osmotin-like protein OSM34	Defense response to fungus	10
3	Q9LJR2	Lectin-like protein LEC	Defense response to fungus, triggered by jasmonate, ethylene and chitin	10
4	O49195	Vegetative storage protein 1	Defense response, response to jasmonic acid	3.4
5	O82122	Vegetative storage protein 2	Defense response to insects; response to wounding, oxidative stress, and jasmonic acid	6.7
6	Q9SR37	Beta-glucosidase 23/ BGLU23/PYK10	Major component of the ER body; glucosinolate catabolic process	10
7	Q9C5C2	Myrosinase 2/Beta-glucosidase 37/ BGLU37	Defense response to insect; glucosinolate catabolic process; role in abscisic acid and methyl jasmonate signaling	2.0
8	Q9SE50	Beta-D-glucopyranosyl abscisate beta-glucosidase/BGLU18	Defense response to fungus and insect; response to salt stress and water deprivation	2.0
9	Q9LJG3	GDSL esterase/lipase ESM1	Response to insect; glucosinolate catabolic process	1.7
10	O04314	PYK10-binding protein 1 (Jacalin-related lectin 30)/PBP1	Regulates the correct polymerization of BGLU23/PYK10 upon tissue damage	10
12	O80948	Jacalin-related lectin 23/JAL23	Polymerization and activation of BGLU23/PYK10 upon tissue damage	3.3
13	O04309	Jacalin-related lectin 35/JAL35	Component of the PYK10 complex; JA-responsive protein	3.0
14	Q9LK72	Lectin-like protein At3g16530	Response to oomycetes	3.2
15	Q9LNN2	Lectin-like protein At1g53070	May be involved in response to insect	2.8
16	Q93XW5	Nitrile-specifier protein 5/NSP5	Glucosinolate catabolic process, nitrile biosynthetic process	4.5
17	Q9M5J9	Polygalacturonase inhibitor 1/PGIP-1	Inhibitor of fungal polygalacturonase. An important factor for plant resistance to phytopathogenic fungi	5.0
18	O81862	Class V chitinase	Hydrolyzes glycol chitin and chitin oligosaccharides; response to abscisic acid and jasmonic acid; response to salt stress	5.1
19	Q9MBH2	Protein AIG2 B / Avirulence-induced gene 2 protein B	Response to bacterium	4.7
20	Q96262	Plasma membrane-associated cation-binding protein 1/PCaP1	Response to oligogalacturonides and flagellin; restricts viral cell-to-cell movement; response to cold, salt, and water deprivation	1.7
21	P32962	Nitrilase 2/NIT2	Involved in plant defense and R gene-mediated resistant responses to microbial pathogens	2.0
**Response to abiotic stress**				
1	Q9SYT0	Annexin D1/ANN1	Response to multiple stresses, including water deprivation	3.8
2	Q0PGJ6	NADPH-dependent aldo-keto reductase, chloroplastic	Response to cold, salt, and water deprivation	10
3	O80944	Aldo-keto reductase family 4 member C8	Response to cold, salt, and water deprivation	1.5
4	O49629	Probable plastid-lipid-associated protein 2, chloroplastic/AtPap2	Probably involved in light/cold stress-related jasmonate biosynthesis	3.8
5	P46422	Glutathione S-transferase F2	Regulation of binding and transport of defense-related compounds during plant stress; binds auxin, flavonoids and camalexin	2.8
6	O80852	Glutathione S-transferase F9	Stress response; detoxification	2.3
7	P42761	Glutathione S-transferase F10	Response to water deprivation	2.0
8	Q9S7E4	Formate dehydrogenase, chloroplastic/mitochondrial/FDH	Oxidoreductase; cell stress response	1.7
9	Q9LZ66	Assimilatory sulfite reductase (ferredoxin), chloroplastic/AtSiR	Sulfate assimilation; response to cold	2.5
**Photosynthesis**				
1	Q9M0V6	Ferredoxin--NADP reductase, root isozyme 1, chloroplastic /RFNR 1	Photosynthesis; ferredoxin-NADP^+^ reductase activity	4.9
2	Q9LHH7	Bifunctional protein FolD 2	Photorespiration; one-carbon metabolic process	2.3
**Oxidative stress and redox homeostasis**				
1	Q9SMU8	Peroxidase 34	Hydrogen peroxide catabolic process; response to oxidative stress; defense response to bacterium and fungus	7.2
2	Q9XEX2	Peroxiredoxin-2B	Cell redox homeostasis, hydrogen peroxide catabolic process	2.3
3	Q9M7T0	Peroxiredoxin-2F, mitochondrial	Cell redox homeostasis; hydrogen peroxide catabolism	1.6
4	Q949U7	Peroxiredoxin-2E, chloroplastic	Cell redox homeostasis, hydrogen peroxide catabolic process	1.6
5	Q9FWR4	Glutathione S-transferase DHAR1, mitochondrial	Key component of the ascorbate recycling system; involved in the redox homeostasis, especially in ROS scavenging under oxidative stress	1.8
6	Q05431	L-ascorbate peroxidase 1, cytosolic	Key role in hydrogen peroxide removal; lignin biosynthetic process; phenylpropanoid biosynthetic process	2.0
7	P42760	Glutathione S-transferase F6	Response to oxidative stress; defense response to bacterium; involved in camalexin biosynthesis; response to water deprivation	5.0
8	Q9ZRW8	Glutathione S-transferase U19	Response to oxidative stress Response to water deprivation	2.0
9	O78310	Superoxide dismutase [Cu-Zn] 2, chloroplastic/CSD2	Removal of superoxide radicals; response to light intensity, UV-B; response to salt stress	2.2
10	P21276	Superoxide dismutase [Fe] 1, chloroplastic	Removal of superoxide radicals. Response to light intensity	1.8
11	P52577	Isoflavone reductase homolog P3	Response to oxidative stress	3.4
12	Q9LSQ5	NAD(P)H dehydrogenase (quinone) FQR1	Response to oxidative stress	2.4
13	Q9SV68	Chloroplast envelope quinone oxidoreductase homolog/ceQORH	Reduces toxic stress-related oxidized lipids produced under oxidative stress conditions	1.8
**Development**				
1	Q9FGY1	Beta-D-xylosidase 1	Seed coat development	4.4
2	O65351	Subtilisin-like protease SBT1.7	Seed coat development	4.7
3	O64530	Thiosulfate/3-mercaptopyruvate sulfurtransferase 1, mitochondrial/MST1	Embryo development ending in seed dormancy	3.9
4	P24806	Xyloglucan endotransglucosylase/hydrolase protein 24	Cell wall biogenesis	4.6
5	P31265	Translationally-controlled tumor protein 1/TCTP1	General regulator required for the development of the entire plant	1.5
**Metabolism**				
1	Q9STT3	Aldose 1-epimerase	Carbohydrate metabolism; glucose metabolic process	10
2	A0A097PMS4	Galactose mutarotase-like superfamily protein	Carbohydrate metabolism	5.0
3	Q9SPK5	Formate--tetrahydrofolate ligase	One-carbon metabolism	4.0
4	Q9LYG3	NADP-dependent malic enzyme 2	Malate and pyruvate metabolic processes; pentose-phosphate shunt, oxidative branch	3.0
5	Q9SK66	NADH dehydrogenase [ubiquinone] 1 alpha subcomplex subunit 9, mitochondrial	Ubiquinone-6 biosynthetic process; electron transport	2.9
6	Q9SMN0	Probable carboxylesterase 12	Carboxylic ester hydrolase activity	3.1
7	Q56WD9	3-ketoacyl-CoA thiolase 2, peroxisomal/ PED1/KAT2	Fatty acid biosynthesis; links fatty acid beta-oxidation with redox regulation; required for the accumulation of benzoylated glucosinolates	3.4
8	O48917	UDP-sulfoquinovose synthase, chloroplastic	Glycolipid and sulfolipid biosynthetic processes	3.0
9	Q8VY84	Probable UMP-CMP kinase1/UMK1	Pyrimidine biosynthesis	2.7
10	P34066	Proteasome subunit alpha type-1-A/PAF1	Ubiquitin-dependent protein catabolic process	2.5
11	Q9XI05	Proteasome subunit beta type-3-A/PBC1	Protein catabolic process	1.7
12	Q56WN1	Glutamine synthetase cytosolic isozyme 1-1	Glutamine biosynthetic process	2.6
13	P47999	Cysteine synthase, chloroplastic/chromoplastic/OASB	A major cysteine synthase	2.2
14	Q9LK23	Glucose-6-phosphate 1-dehydrogenase 5, cytoplasmic	Pentose-phosphate shunt, oxidative branch	2.1
15	Q9FIE8	UDP-glucuronic acid decarboxylase 3	UDP-D-xylose biosynthetic process	1.9
16	Q9S9W2	Short-chain dehydrogenase/reductase SDRA	Fatty acid metabolic process	1.6
17	Q9T034	Phenylalanine--tRNA ligase alpha subunit, cytoplasmic	Protein biosynthesis	1.5
18	Q8L934	Nucleoid DNA-binding-like protein	Proteolysis	10
**Other proteins**				
1	Q9ZSK4	Actin-depolymerizing factor 3/ADF-3	Actin filament depolymerization	4.7
2	Q29Q34	NAD(P)-binding Rossmann-fold superfamily protein / At5g19440	Oxidoreductase activity; response to *Agrobacterium tumefaciens*	1.5
3	O23016	Probable voltage-gated potassium channel subunit beta	Potassium ion transport	1.9
4	Q9XI10	DPP6 N-terminal domain-like protein	Unknown function	4.7
5	Q8GYZ3	RmlC-like jelly roll fold protein	Unknown function	3.1
6	Q9SSK5	MLP-like protein 43	Unknown function	3.0

Notes: ^1^ Protein function is presented according to the UniProtKB and TAIR databases. ^2^ Mean of three biological repeats. The table shows proteins with statistically significant differences from the proteins of WT plants (*p* < 0.05, Student’s *t*-test). Fold changes in protein expression were assessed based on the mean protein intensity using PDQuest 8.0.1 Advanced software (see Section 4).

**Table 2:** Proteins downregulated in *rolB*-expressing *Arabidopsis* plants.

	UniProtKB Code	Name of the Protein	Protein Function ^1^	Inhibition, Folds ^2^
1	O24456	Receptor for activated C kinase 1A/ RACK1A	Major component of the RACK1 regulatory proteins that play a role in multiple signal transduction pathways	1.5
2	Q9C4Z6	Receptor for activated C kinase 1B/RACK1B	Minor component of the RACK1 regulatory proteins	3.8
3	Q9LV28	Receptor for activated C kinase 1C /RACK1C	Minor component of the RACK1 regulatory proteins	4.2
**Chaperones**				
1	Q9LF37	Chaperone protein ClpB3, chloroplastic	Molecular chaperone essential for chloroplast development and seedling viability; response to heat	3.0
2	P42730	Chaperone protein ClpB1/Heat shock protein 101	Molecular chaperone that plays an important role in thermotolerance	3.0
3	Q9FI56	Chaperone protein ClpC1, chloroplastic	Regulation of chlorophyll biosynthetic process; chloroplast organization	3.1
4	Q9SAR5	Ankyrin repeat domain-containing protein 2A	Chaperone; protein targeting to chloroplast	2.2
5	Q03250	Glycine-rich RNA-binding protein 7/AtGR-RBP7/ GRP7	Chaperone; innate immunity; plant defense; target of the *Pseudomonas syringae* type III effector HopU1	2.0
6	P42763	Dehydrin ERD14	Chaperone, protein stabilization; response to cold and water deprivation	1.9
7	Q9LNB6	Hsp70-Hsp90 organizing protein 1/AtHop1	Mediates the association of the molecular chaperones HSP70 and HSP90; stress response	1.5
**Plant defense**				
1	Q9FN05	Probable glucan 1,3-alpha-glucosidase	Defense response to bacterium; required for sustained activation of EFR-mediated signaling	3.1
2	Q9FXA2	Polyadenylate-binding protein 8/PABP-8	Host-virus interaction	1.7
3	Q9ZUU4	RNA-binding protein CP29B, chloroplastic	Innate immune response; potential target of HopU1	1.7
4	Q9FGS0	RNA-binding protein CP31B, chloroplastic	Innate immune response; potential target of HopU1	1.7
**Response to stress**				
1	P42759	Dehydrin ERD10	Protein stabilization; cold acclimation; response to abscisic acid and water deprivation	3.9
2	P54887	Delta-1-pyrroline-5-carboxylate synthase A	Plays a key role in proline biosynthesis. Response to abscisic acid, cold, desiccation, salt stress, and oxidative stress	2.4
3	Q9FLT0	Ribonuclease TUDOR 2	Cytoprotective ribonuclease, which is essential for resistance to abiotic stress	2.3
4	O23523	RGG repeats nuclear RNA binding protein A	Promotes stomata closure in drought conditions; involved in resistance to salt and drought	1.5
**Photosynthesis**				
1	Q9SW18	Magnesium protoporphyrin IX methyltransferase, chloroplastic	Chlorophyll biosynthesis	3.0
2	Q9LR75	Coproporphyrinogen-III oxidase 1, chloroplastic	Chlorophyll biosynthesis	5.0
3	O22886	Uroporphyrinogen decarboxylase 2, chloroplastic	Chlorophyll biosynthesis	2.8
4	P21218	Protochlorophyllide reductase B, chloroplastic	Chlorophyll biosynthesis	2.4
5	O48741	Protochlorophyllide reductase C, chloroplastic	Chlorophyll biosynthesis	2.4
6	Q9FMD5	Protein TIC 40, chloroplastic	Chloroplast organization, protein import into chloroplast stroma	2.3
7	Q9FKW6	Ferredoxin--NADP reductase, leaf isozyme 1, chloroplastic/FNR 1	Photosynthetic electron transport chain	2.0
8	Q8W493	Ferredoxin--NADP reductase, leaf isozyme 2, chloroplastic /FNR 2	Regulates photosynthetic electron flow during the transition from dark to light	1.7
9	Q01908	ATP synthase gamma chain 1, chloroplastic/ATPC1	ATP biosynthetic process; photosynthetic electron transport in photosystem II	1.8
10	P42699	Plastocyanin major isoform, chloroplastic	Electron transfer between P700 and the cytochrome b6-f complex in photosystem I	3.0
11	Q9ZR03	Cytochrome b6-f complex iron-sulfur subunit, chloroplastic	Electron transfer between photosystem II and photosystem I	1.5
12	P37107	Signal recognition particle 54 kDa protein, chloroplastic	Required for light-harvesting chlorophyll a/b-binding protein integration into thylakoids	4.3
13	P27140	Beta carbonic anhydrase 1, chloroplastic/AtbCA1	Photosynthesis; carbon utilization	3.6
14	O80796	Membrane-associated protein VIPP1, chloroplastic	Thylakoid membrane organization	1.9
**General metabolism**				
1	P42737	Beta carbonic anhydrase 2, chloroplastic/AtbCA2	Reversible hydration of carbon dioxide; plays an important role in amino acid biosynthesis	9.8
2	Q9FYE3	Alpha carbonic anhydrase 3/AtaCA3	Reversible hydration of carbon dioxide	2.5
3	Q9LR30	Glutamate--glyoxylate aminotransferase 1	Glycine biosynthetic process	2.2
4	Q56YA5	Serine-glyoxylate aminotransferase	Glycine biosynthetic process	1.9
5	Q94B78	Glycine dehydrogenase (decarboxylating) 1, mitochondrial	Glycine catabolic process	2.8
6	Q8W593	Probable lactoylglutathione lyase, chloroplastic	Methylglyoxal catabolic process; response to cold	3.3
7	O50008	5-methyltetrahydropteroyltriglutamate homocysteine methyltransferase 1	Methionine biosynthesis	3.0
8	Q9SYM5	Trifunctional UDP-glucose 4,6-dehydratase/UDP-4-keto-6-deoxy-D-glucose 3,5-epimerase/UDP-4-keto-L-rhamnose-reductase RHM1	UDP-rhamnose biosynthetic process; plays a major role in supplying UDP-rhamnose for flavonol biosynthesis	1.7
9	P56757	ATP synthase subunit alpha, chloroplastic	ATP biosynthesis; response to cold	3.0
10	Q9C7N5	GDSL esterase/lipase At1g29660	Lipid metabolism	3.0
11	O23553	Beta-amylase 3, chloroplastic	Carbohydrate metabolism; response to cold	3.0
12	Q84TF0	Aldo-keto reductase family 4-member C10	Metabolism of ketosteroids and aldehydes	1.5
13	Q9LD43	Acetyl-coenzyme A carboxylase carboxyl transferase subunit alpha, chloroplastic	Fatty acid biosynthesis	2.5
14	P52410	3-oxoacyl-[acyl-carrier-protein] synthase I, chloroplastic	Fatty acid biosynthesis	1.5
15	P42734	Probable cinnamyl alcohol dehydrogenase 9	Lignin biosynthesis	1.7
16	O64767	AICARFT/IMPCHase bienzyme family protein	Purine biosynthesis	1.5
17	Q96533	Alcohol dehydrogenase class-3	Ethanol oxidation; formaldehyde catabolic process	1.6
18	Q949Y0	Ubiquitin carboxyl-terminal hydrolase 6	Proteasome-mediated ubiquitin-dependent protein catabolic process	3.4
19	Q8H0S9	Puromycin-sensitive aminopeptidase/PSA	Proteolysis; essential for cell growth and viability	3.0
20	Q1EBV4	Protein DNA-DAMAGE INDUCIBLE 1	Proteolysis	2.3
**Protein biosynthesis**				
1-2	P49227 Q8LBI1	60S ribosomal protein L5-2 60S ribosomal protein L5-1	Protein biosynthesis; leaf morphogenesis; root morphogenesis	2.3
3-4	Q9SIP7 Q9FJA6	40S ribosomal protein S3-1 40S ribosomal protein S3-3	Translation	2.3
5	Q93VC7	30S ribosomal protein S1, chloroplastic	Translation; required for optimal plastid performance in terms of photosynthesis	1.8
6	O50061	50S ribosomal protein L4, chloroplastic	Translation	1.7
7	P36210	50S ribosomal protein L12-1, chloroplastic	Translation	1.6
8	P51412	50S ribosomal protein L21, chloroplastic	Translation; embryo development ending in seed dormancy	1.8
9	Q9LY66	50S ribosomal protein L1, chloroplastic	Translation	1.5
10	Q9SI75	Elongation factor G, chloroplastic	Protein biosynthesis; post-embryonic development	2.3
11	Q9ASR1	Elongation factor 2	Protein biosynthesis; cold acclimation	2.1
12	Q8VZW6	Elongation factor P (EF-P) family protein	Protein biosynthesis	2.0
**Other proteins**				
1	Q941D3	Probable plastid-lipid-associated protein 8, chloroplastic	Unknown function	2.4
2	Q8L606	Tetratricopeptide repeat (TPR)-like superfamily protein	Unknown function	2.9
3	Q9LVT8	RGG repeats nuclear RNA binding protein C	Unknown function	1.5

Notes: ^1^ Protein function is presented according to the UniProtKB and TAIR databases. ^2^ Mean of three biological repeats. The table shows proteins with statistically significant differences from the proteins of control plants (P < 0.05, Student’s *t*-test). Fold changes in protein expression were assessed based on mean protein intensity using PDQuest 8.0.1 Advanced software (see Section 4).

**Table 3:** RACK1A-interacting proteins which abundance was changed in *rolB*-plants, line AtB-1.

UniProtKB Code	Protein	Function or Biological Process	Reference
**Upregulated**			
Q42406	Peptidyl-prolyl cis-trans isomerase CYP18-4/Rotamase cyclophilin-5/ROC5	The closest analogue AtCYP18-3 is involved in plant-pathogen interactions	[56]
Q9ZRW8	Glutathione S-transferase U19, cytosolic	Response to oxidative stress	[57]
O49195	Vegetative storage protein1/VSP1	Defense response. Induced by mechanical wounding, jasmonic acid (JA), insect herbivory, osmotic and nutritional stresses	[40]
Q93XW5	Nitrile-specifier protein 5	Defense against herbivory and pathogen attacks; component of the glucosinolate-myrosinase system	[58]
Q9LK72	Lectin-like protein At3g16530	Plant defense response; homologs are induced by fungal elicitors	[59]
Q9SR37	Beta-glucosidase 23/PYK10	Defense against pests and fungi	[33]
Q9S7E4	Formate dehydrogenase, chloroplastic/mitochondrial	Formaldehyde metabolism; induced by various stresses	[60]
P21276	Superoxide dismutase [Fe] 1, chloroplastic/FSD1	Response to oxidative stress: response to light intensity	[61]
Q9M5J9	Polygalacturonase inhibitor 1/ PGIP1	Restricts the growth of invasive fungal pathogens; defense against cyst nematodes	[41,42]
Q05431	L-ascorbate peroxidase 1, cytosolic	Cellular response to oxidative stress; phenylpropanoid biosynthetic process; lignin biosynthesis	[62]
P24806	Xyloglucan endotransglucosylase/ hydrolase protein 24	Cell wall biogenesis	[63]
**Downregulated**			
P27140	Beta carbonic anhydrase 1, chloroplastic	Photosynthesis; stomatal development; defense response	[64]
P36210	50S ribosomal protein L12-1, chloroplastic	Translation	UniProtKB
Q9LVT8	RGG repeats nuclear RNA binding protein C	mRNA and RNA binding	UniProtKB
O80796	Membrane-associated protein VIPP1, chloroplastic	Thylakoid membrane organization and vesicle organization	[65]

## Data Availability

The mass spectrometry data have been deposited to ProteomeXchange via the PRIDE partner repository with the dataset identifier PXD037959 (Project DOI: 10.6019/PXD037959).

## Supplementary Materials

The following supporting information can be downloaded at: https://www.mdpi.com/article/10.3390/ijms24031880/s1. References are cited in [103,104,105,106].
