# Supplementary material for: Proteomic Analysis of Proteins Related to Defense Responses in Arabidopsis Plants Transformed with the rolB Oncogene
Source: Int J Mol Sci. 2023 Jan 18;24(3):1880. doi: 10.3390/ijms24031880 (PMC9915171; doi:10.3390/ijms24031880)
Supplement: Supplementary file 1 [file ijms-24-01880-s001.zip › ijms-2108519-supplementary.pdf]

SUPPLEMENTARY INFORMATION

Proteomic analysis of proteins related to defense responses in *Arabidopsis* plants transformed with the *rolB* oncogene

Yulia V. Vereshchagina[a], Anastasiya A. Mironova[a], Dmitry V. Bulgakov[a], Victor P. Bulgakov[a,*]

[a]Federal Scientific Center of the East Asia Terrestrial Biodiversity, Far East Branch of Russian Academy of Sciences, 159 Stoletija Str., Vladivostok, 690022, Russia

Supplementary Table S1. Sequences of the PCR primers used in this study
Supplementary Table S2. Comparison of protein expression and expression of the corresponding genes
Supplementary Figure S1. Standard curve for absolute quantification of *rolB* expression.
Supplementary Figure S2. Induction of proteins of the PYK10 complex by the *rolB* gene.

Supplementary Table S1. Sequences of the PCR primers used in this study

UniProtKB code	Genbank accession number	Primer name	Primer sequences (5'-3')	Ref.
P20402 (ROB1_AGRRH)	X03433	*rolB*-780-D	ATGGATCCCAAATTGCTATTC	[1]
		rolB-780-R	TTAGGCTTCTTTCTTCAGGTT	
		rolB-D	ACATCATAGGGGCGGTTTTCAGT	[2]
		rolB-R	TTTCGCAAGTTCCTTGTTCATTC	
Q84K90 (RHIP1_ARATH)	BT002964	*RHIP1*-D	GAGCTGAAGTGGCTTCAATGAC	[3]
		RHIP1-R	GGTCCGACATACCCATGATCC	
O24456 (GBLPA_ARATH)	AY035007	*RACK1A*-D	GCTGAAAAGGCTGACAACAGT	[4]
		RACK1A-R	GCTCCAGTTAAGGCTTGTGC	
Q9C4Z6 (GPLPB_ARATH)	AY059723	*RACK1B*-D	TGTTGAGGATTTGAAGGTTGA	
		RACK1B-R	CCAGTTCAAGCTTGTGCAGTA	
Q9LV28 (GPLPC_ARATH)	AY050338	*RACK1C*-D	GAGGCAGAGAAGAATGAAGGTG	
		RACK1C-R	CCAGTTCAAGCTTGTGCAGTA	
P34790	AY093227	*CYP18-3*-D	GAACGGAACAGGCGGTGAG	

(CP18C_ARATH)		*CYP18-3*-R	TCAAGCCAATCGGTCTTCACG	
Q38867 (CP19C_ARATH)	AY072128	*CYP19-3*-D	CTTTCACCGTATAATCCCAG	[5]
		CYP19-3-R	CCTGTCAAGATCAACCCACCC	
Q38900 (CP19A_ARATH)	AY048215	*CYP19-1*-D	GGTCAAGTTGTTGAGGGATTG	
		CYP19-1-R	ACACAAAGCTACCATTGGATC	
P34791 (CP20C_ARATH)	AF325026	*CYP20-3*-D	CAATGCATCGGTCAATAGTG	[5]
		CYP20-3-R	CTGGTGAGAAGAAATACGGG	
Q42406 (CP18D_ARATH)	AY054468	*CYP18-4*-D	ATCTACGGTGCTAAGTTCAAGG	
		CYP18-4-R	GTGCTTACCATCTAACCACGAC	
A8R7E6 (CERK1_ARATH)	AB367524	*CERK1*-D	TTATAGGAGTGATTGTGGCTTTG	
		CERK1-R	CCTTAGTAGACAACGGAATAGAAGA	
Q39023 (MPK3_ARATH)	AF386961	*MPK3*-D	GCCCTTAGCTAAACTTTTCTC	[6]
		MPK3-R	CGTGCAATTTAGCAAGGTACT	
Q39024 (MPK4_ARATH)	AF360231	*MPK4*-D	AGCAGACGCATCACAGTTGA	
		MPK4-R	TGAACGGCCTCACACATACC	
Q39026 (MPK6_ARATH)	AY120737	*MPK6*-D	CGTTTGTTCGGCTATGAATTCTG	[7]
		MPK6-R	GTGGCGGGATAATATCTCTGATTG	
Q9XI87 (Q9XI87_ARATH)	BT026491	*VIK*-D	ATGGCTCCTGAAGTATTCAAGC	[8]
		VIK-R	TCTTGAGAATGTCCAGAAACGACG	
P43082 (HEVL_ARATH)	AF370536	*HEL*-D	TGTTCTCCGACCAACAACTG	
		HEL-R	GTTCTTCACCCTTAAACACTTGC	
O49195 (VSP1_ARATH)	AF386930	*VSP1*-D	CATCTCATACTCAAGCCAAACG	
		VSP1-R	AGTATCCTCAACCAAATCAGC	
O82122 (VSP2_ARATH)	AY048282	*VSP2*-D	ATGGATACGGAACAGAGAAGACC	
		VSP2-R	CGAGAGTGACATTCTTCCACAAC	
Q9SR37 (BGL23_ARATH)	AF386967	*BGLU23* -D	CGTCAATGCTAAATGCCAAGA	
		BGLU23 -R	CGAACCAAGCAGGACTATGTG	
O04314	AF370488	*PBP1*-D	TACCAAAGGCCGTGTTCTCC	

(JAL30_ARATH)		*PBP1*-R	CATCCCATACAGTTCCGTCATC	
Q03250 (RBG7_ARATH)	AF428381	*RBG7*-D	GCGACGTTATTGATTCCAAG	
		RBG7-R	TTGTCCGTTCATTCCCTCA	
Q9LKR3 (BIP1_ARATH)	BT000453	*BIP1/2*-D	TCACTTGGGAGGTGAGGACTTT	[9]
Q39043 (BIP2_ARATH)	BT002392	*BIP1/2*-R	CTCACATTCCCTTCGGAGCTTA	
Q9LTX9 (HSP7G_ARATH)	BT000919	*HSP70-7*-D	CAACTCAAGAAATCAAAGACAC	[5]
		HSP70-7-R	CGTCAATCACATCACCGCCG	
Q9SIF2 (HS905_ARATH)	AF436826	*HSP90-5*-D	CTTCTCCTTGTGTGCTTGTC	
		HSP90-5-R	CTTGTTGCTTCAGTGCTCTC	
O65282 (CH10C_ARATH)	AJ010818	*CPN20*-D	CCGAGACTACCAAAGAGAAG	
		CPN20-R	CTAAGAAAGTATAGCCATCAC	

Supplementary Table S2. Comparison of protein expression and expression of the corresponding genes; data from the present study and previous study for *rolB* transformed calli.

Protein Name	UniProtKB code	***rolB*-plants**		***rolB*-calli**
		Protein (2DE)	*mRNA (qPCR)*	*Protein (2DE)* [5]
RACK1-associated proteins				
RACK1A	O24456 (GBLPA_ARATH)	**1,48* down**	**1,44** down**	**1,5* down**
RACK1B	Q9C4Z6 (GPLPB_ARATH)	**3,8* down**	**1,7* down**	Not found
RACK1C	Q9LV28 (GPLPC_ARATH)	**4,2* down**	**3,55* down**	Not found
Defense reactions and plant immunity				
PBP1 (JAL30)	O04314 (JAL30_ARATH)	**10* up**	**2,18* up**	Not found
PYK10 (BGL23)	Q9SR37 (BGL23_ARATH)	**10* up**	**2,24* up**	Not found
GRP7	Q03250 (RBG7_ARATH)	**1,98* down**	**3,31* down**	Not found
HEVL	P43082 (HEVL_ARATH)	**10* up**	**9,04* up**	Not found
VIK	Q9XI87 (Q9XI87_ARATH)	Not found	1,03 down	**2* down**
VSP1	O49195 (VSP1_ARATH)	**3,4* up**	**2,73** up**	Not found

VSP2	O82122 (VSP2_ARATH)	**6,7* up**	**2,58** up**	Not found
CERK1-associated proteins.				
CERK1	A8R7E6 (CERK1_ARATH)	Not found	1,05 up	Not found
MPK3	Q39023 (MPK3_ARATH)	Not found	1,06 down	Not found
MPK4	Q39024 (MPK4_ARATH)	Not found	1,43 up	Not found
MPK6	Q39026 (MPK6_ARATH)	Not found	1,28 up	Not found
Chaperone-type proteins.				
BIP1/2	Q9LKR3 (BIP1_ARATH)<br>Q39043 (BIP2_ARATH)	**2,8* up**	**2,55* up**	Not found
HSP70-7	Q9LTX9 (HSP7G_ARATH)	Not found	1,104 up	**2.9* up**
HSP90-5	Q9SIF2 (HS905_ARATH)	Not found	1,13 up	**2.4* up**
CPN10	O65282 (CH20_ARATH)	Not found	**2,43* up**	**2* up**
ROC1 (CYP18-3)	P34790 (CP18C_ARATH)	**2,0* up**	**1,84* up**	**6,6* down**
ROC2 (CYP19-3)	Q38867 (CP19C_ARATH)	**3,8* up**	**1,65* up**	**10* down**
ROC3 (CYP19-1)	Q38900 (CP19A_ARATH)	**1,4* up**	**1,41* up**	Not found
ROC4 (CYP20-3)	P34791 (CP20C_ARATH)	Not found	**4,96* down**	**10* down**
ROC5 (CYP18-4)	Q42406 (CP18D_ARATH)	**1,7* up**	**1,72* up**	Not found

Asterisks indicate statistically significant differences of means (*P <0.05; **P <0.01), Fisher's LSD.

References

1. Bettini, P.P.; Marvasi, M.; Fani, F.; Lazzara, L.; Cosi, E.; Melani, L.; Mauro, M.L. *Agrobacterium rhizogenes rolB* gene affects photosynthesis and chlorophyll content in transgenic tomato (*Solanum lycopersicum L.*) plants. *J. Plant. Physiol.* ***2016****, 204*, 27-35, doi: 10.1016/j.jplph.2016.07.010.
2. Grishchenko, O.V.; Kiselev, K.V.; Tchernoded, G.K.; Fedoreyev, S.A.; Veselova, M.V.; Bulgakov, V.P.; Zhuravlev, Y.N. *RolB* gene-induced production of isoflavonoids in transformed *Maackia amurensis* cells. *Appl. Microbiol. Biotechnol.* **2016**, *100*, 7479-7489, doi: 10.1007/s00253-016-7483-y.
3. Brzezinka, K.; Altmann, S.; Czesnick, H.; Nicolas, P.; Gorka, M.; Benke, E.; Kabelitz, T.; Jähne, F.; Graf, A.; Kappel, C.; Bäurle, I. *Arabidopsis* FORGETTER1 mediates stress-induced chromatin memory through nucleosome remodeling. *Elife* **2016**, *5, e17061*, doi: 10.7554/eLife.17061.
4. Cheng, Z.; Li, J.F.; Niu, Y.; Zhang, X.C.; Woody, O.Z.; Xiong, Y.; Djonović, S.; Millet, Y.; Bush, J.; McConkey, B.J.; Sheen, J.; Ausubel, F.M. Pathogen-secreted proteases

activate a novel plant immune pathway. *Nature* **2015**, *521*, 213-216, doi: 10.1038/nature14243.

5. Bulgakov, V.P.; Vereshchagina, Y.V.; Bulgakov, D.V.; Veremeichik, G.N.; Shkryl, Y.N. The rolB plant oncogene affects multiple signaling protein modules related to hormone signaling and plant defense. *Sci. Rep.* **2018**, *8*, 2285, doi: 10.1038/s41598-018-20694-6.
6. Ye, L.; Li, L.; Wang, L.; Wang, S.; Li, S.; Du, J.; Zhang, S.; Shou, H. MPK3/MPK6 are involved in iron deficiency-induced ethylene production in *Arabidopsis*. *Front. Plant Sci.* **2015**, 6, 953, doi: 10.3389/fpls.2015.00953.
7. Zhao, L.; Wang, C.; Zhu, F.; Li, Y. Mild osmotic stress promotes 4-methoxy indolyl-3-methyl glucosinolate biosynthesis mediated by the MKK9-MPK3/MPK6 cascade in *Arabidopsis*. *Plant Cell. Rep.* **2017**, 36, 543-555, doi: 10.1007/s00299-017-2101-8.
8. Wingenter, K.; Trentmann, O.; Winschuh, I.; Hörmiller, I.I.; Heyer, A.G.; Reinders, J.; Schulz, A.; Geiger, D.; Hedrich, R.; Neuhaus, H.E. A member of the mitogen-activated protein 3-kinase family is involved in the regulation of plant vacuolar glucose uptake. *Plant J.* **2011**, *68*, 890-900, doi: 10.1111/j.1365-313X.2011.04739.x.
9. Arraño-Salinas, P.; Domínguez-Figueroa, J.; Herrera-Vásquez, A.; Zavala, D.; Medina, J.; Vicente-Carbajosa, J.; Meneses, C.; Canessa, P.; Moreno, A.A.; Blanco-Herrera, F. WRKY7, -11 and -17 transcription factors are modulators of the bZIP28 branch of the unfolded protein response during PAMP-triggered immunity in *Arabidopsis thaliana*. *Plant Sci.* **2018**, *277*, 242-250, doi: 10.1016/j.plantsci.2018.09.019.

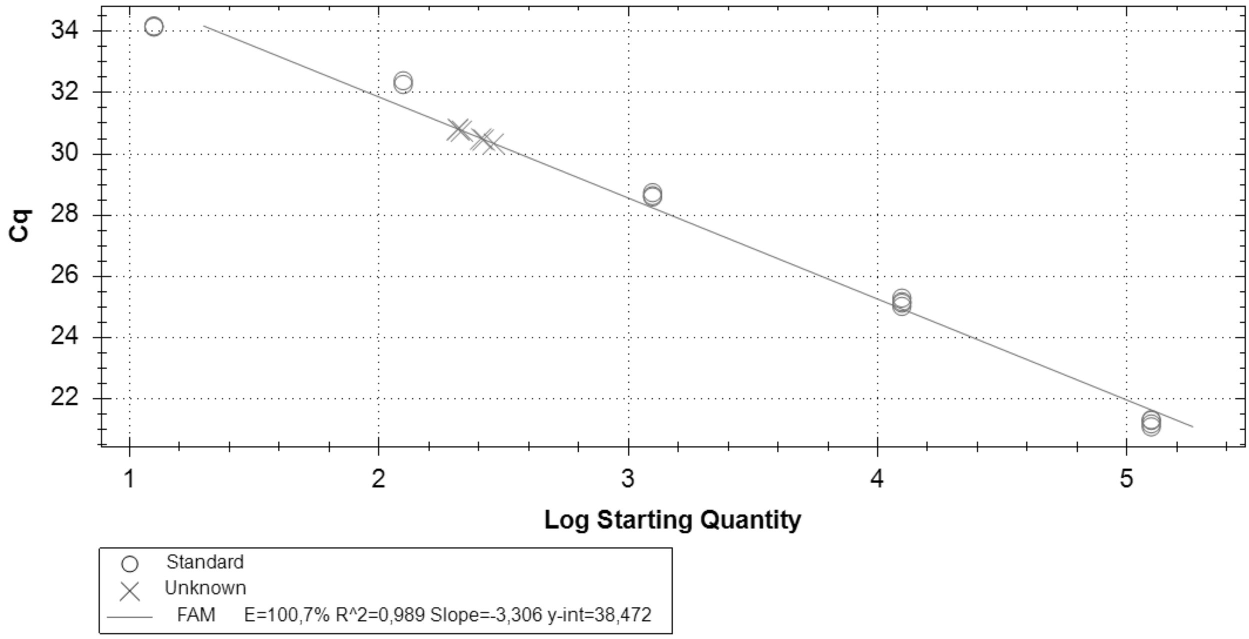

Supplementary Figure S1. Standard curve for absolute quantification of *rolB* expression. Standard curve was constructed using 1.25×10^1 to 1.25×10^5 copies/µl of *rolB* amplicon (three technical replicates for each standard dilution). The data represent a result from two biological replicates with cDNA samples (marked by a cross) analyzed in triplicate.

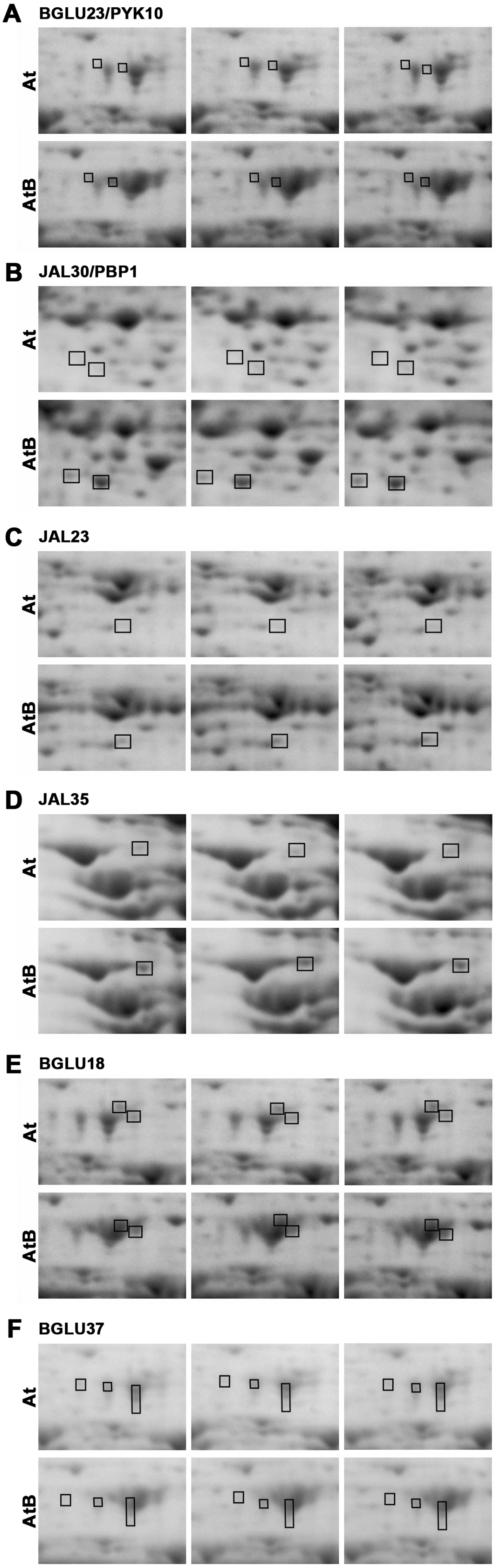
A BGLU23/PYK10
At
AtB
B JAL30/PBP1
At
AtB
C JAL23
At
AtB
D JAL35
At
AtB
E BGLU18
At
AtB
F BGLU37
At
AtB

Supplementary Figure S2. Induction of proteins of the PYK10 complex by the *rolB* gene. Fragments of 2D gels of the control (At) and *rolB* AtB-1 (AtB) plant protein fractions are presented. **A-F**: beta-glucosidases 23 (PYK10), PYK10-binding protein 1 (jacalin-related lectin 30)/PBP1, jacalin-related lectin 23, jacalin-related lectin 35, beta-D-glucopyranosyl abscisate beta-glucosidase/BGLU18, and myrosinase 2/Beta-glucosidase 37/ BGLU37
